# Quality Improvement Initiative to Decrease Variability of Emergency Physician Opioid Analgesic Prescribing

**DOI:** 10.5811/westjem.2016.3.29692

**Published:** 2016-05-02

**Authors:** John H. Burton, Jason A. Hoppe, Jeff M. Echternach, Justin M. Rodgers, Michael Donato

**Affiliations:** * Carilion Clinic, Department of Emergency Medicine, Roanoke, Virginia; † University of Colorado Denver School of Medicine, Department of Emergency Medicine, Aurora, Colorado; ‡ Rocky Mountain Poison and Drug Center, Denver, Colorado

## Abstract

**Introduction:**

Addressing pain is a crucial aspect of emergency medicine. Prescription opioids are commonly prescribed for moderate to severe pain in the emergency department (ED); unfortunately, prescribing practices are variable. High variability of opioid prescribing decisions suggests a lack of consensus and an opportunity to improve care. This quality improvement (QI) initiative aimed to reduce variability in ED opioid analgesic prescribing.

**Methods:**

We evaluated the impact of a three-part QI initiative on ED opioid prescribing by physicians at seven sites. Stage 1: Retrospective baseline period (nine months). Stage 2: Physicians were informed that opioid prescribing information would be prospectively collected and feedback on their prescribing and that of the group would be shared at the end of the stage (three months). Stage 3: After physicians received their individual opioid prescribing data with blinded comparison to the group means (from Stage 2) they were informed that individual prescribing data would be unblinded and shared with the group after three months. The primary outcome was variability of the standard error of the mean and standard deviation of the opioid prescribing rate (defined as number of patients discharged with an opioid divided by total number of discharges for each provider). Secondary observations included mean quantity of pills per opioid prescription, and overall frequency of opioid prescribing.

**Results:**

The study group included 47 physicians with 149,884 ED patient encounters. The variability in prescribing decreased through each stage of the initiative as represented by the distributions for the opioid prescribing rate: Stage 1 mean 20%; Stage 2 mean 13% (46% reduction, p<0.01), and Stage 3 mean 8% (60% reduction, p<0.01). The mean quantity of pills prescribed per prescription was 16 pills in Stage 1, 14 pills in Stage 2 (18% reduction, p<0.01), and 13 pills in Stage 3 (18% reduction, p<0.01). The group mean prescribing rate also decreased through each stage: 20% in Stage 1, 13% in Stage 2 (46% reduction, p<0.01), and 8% in Stage 3 (60% reduction, p<0.01).

**Conclusion:**

ED physician opioid prescribing variability can be decreased through the systematic application of sharing of peer prescribing rates and prescriber specific normative feedback.

## INTRODUCTION

Pain is a major reason for patients to seek care in the emergency department (ED), accounting for approximately 42% of ED visits.^1^–^3^ Emergency physicians have been scrutinized for inadequately addressing analgesia.^3^–^6^ National pain treatment initiatives have contributed to a substantial increase in the prescribing of opioid analgesics in the United States. From 1999 to 2008, retail sales of opioid analgesics nearly doubled; unfortunately, prescription opioid abuse and overdose rates have paralleled this rise in opioid prescribing.^7^ Deaths in the U.S. attributed to prescription opioid overdose have surpassed deaths due to motor vehicle crashes as the leading cause of accidental death.^8^

Much of the dialogue regarding possible solutions to the epidemic of opioid abuse and overdose includes addressing the physician’s role in prescribing opioids. The decision as to whether opioids are necessary, appropriate, and safe is complex. In their contemplation of how to address pain, healthcare providers attempt to balance the patient’s needs and goals with the benefits and potential side effects of available treatments. Despite the heightened awareness of harm from opioids and recent interventions, physician opioid prescribing practices remain highly variable.^7^,^9^,^10^ Tamayo-Sarver et al. found the decision to prescribe opioids to be very inconsistent, even when physicians are provided identical patient scenarios. Moreover, respondents in this investigation reported that the same clinical information (e.g. a patient requesting a strong analgesic) changed the likelihood of prescribing opioids in opposite directions for different physicians.^11^

It is not known whether providing objective feedback to physicians regarding their own opioid analgesic prescribing and that of their peers can decrease the variability of opioid prescribing within an ED practice environment. To our knowledge there have been no studies assessing the effect of quality improvement (QI) initiatives on physician opioid prescribing practices. To address this question, we used electronic health record (EHR) prescription information to generate aggregate and prescriber-specific data for our ED physician group. During the implementation of a QI project, we measured the impact of using sequential feedback of this prescribing data on the variability of opioid prescribing within the group. We hypothesized that sharing of peer opioid-prescribing rates with normative feedback to physicians would decrease prescribing variability among an emergency medicine physician group.

## METHODS

### Study Design and Setting

This QI initiative was implemented in a large, hospital-employed physician group staffing seven EDs with approximately 265,000 combined annual visits. The setting included a variety of practice locations including both academic and private as well as urban and rural locations. All sites share a common EHR (Epic 2010 Verona, WI). All prescriptions are ordered electronically via the EHR. The local institutional review board (IRB) determined this project was QI and waived further IRB oversight.

### Subjects

The physician group includes both EM boarded and non-EM boarded physicians working fulltime in the seven EDs. Pediatric EM physicians, mid-level providers and residents were excluded. We included for analysis all opioid prescriptions written at ED discharge over the 15-month data collection period from February 2012 to April 2013. Prior to study initiation, an institution-specific controlled medication prescription policy and an Internet-based statewide prescription drug monitoring program (PDMP) existed. The institution specific policy was instituted in 2009 and was identical across all practice sites with no changes during the data collection period for this project. The PDMP in our state includes mandated pharmacist entry for all controlled substance prescriptions at the time the prescription is filled. The PDMP was accessible to all physicians in the ED practice group and it remained unchanged with regards to entry of patients and prescriber access during the data collection period. There was no formal policy in the physician practice group or at a state level that structured or directed use of this program. Therefore, physicians used this database at their own discretion with influences on their prescribing patterns unique to each physician.

### Methods and Measurements

We abstracted clinical data for all ED discharges from the EHR via a computer algorithm. These included visit date opioid analgesic prescriptions (medication and quantity of pills), and ED provider identity. Tramadol and liquid cough preparations were excluded from the analysis a priori as the focus was on the most common prescription opioids. No patient identifiers (medical record number or patient identity) were recorded in the database or reported to the prescribing physicians.

This QI project incorporated three sequential stages. Study stage 1 was a nine-month baseline period during which physicians were unaware of the evaluation or planned report for this stage or future stages. The project lead investigator was the only physician aware of the data gathering, the intention to perform a QI intervention, and the initial results. In stage 2, physicians were informed via physician practice meetings and protected institutional email of the following: (1) a three-month prospective opioid prescribing report would be collected for all ED discharges; (2) prescribing data would be tracked; and (3) the intent of the initiative was to inform physicians of their practices and practice group mean opioid-prescribing rates. Physicians were informed that they would privately receive their own data at the conclusion of this period and that there would be no punitive use of the data. They were unaware of the baseline data set.

At the conclusion of stage 2 each ED physician received their own data via email demonstrating their individual rate of opioid analgesic prescribing. Each physician also received the calculated mean rates of opioid prescribing for discharged ED patients for their peer groups, both at their specific practice site and all other practice sites in the physician group. Therefore, all physicians were aware of their own frequency of prescribing opioids at discharge, but remained blinded to all other individuals in the group except for the means for each practice site and the group mean. Lastly, physicians were informed via the same methods (meetings and email) that a second three-month interval (stage 3) would begin after their receipt of the stage 2 data report.

In stage 3, the same data would be collected for each physician using the same methods. Physicians were informed that at the completion of this stage, they would again receive their personal opioid prescribing rates and the group means both overall and at each site. In addition, each physician would also receive the unblinded data results for each physician in the group.

### Outcomes

The primary outcome goal of the QI project was to decrease variability of the physician group’s opioid prescribing for discharged ED patients. This process endpoint was chosen given that QI projects that improve system defects have the potential to maximize the effect on the care of all patients.^12^ Group prescribing variability was measured with sequential interventions, both when physicians became aware of their own frequency of prescribing relative to their site and group practice mean (stage 2) and when they were aware that unblinded peer physician-prescribing data would be provided (stage 3). We hypothesized that transparency of prescribing behaviors with provider-specific feedback would decrease variability in physician frequency of opioid prescribing, resulting in the physician group normalizing around the group mean during sequential assessments. Secondary outcomes for each stage were changes in opioid prescribing frequency and changes in the quantity of pills written per opioid analgesic prescription.

The calculation of our opioid analgesic prescription reporting metrics was straightforward. Two metrics were calculated based on the data collected: (1) opioid prescribing rate, defined as the number of patients discharged from the ED with opioid prescriptions divided by total number of patient discharge encounters for each individual prescriber, and (2) opioid pill count, defined as the mean quantity of opioid analgesic pills written per prescription. Using these two metrics, we were able to generate both physician specific and aggregate data for each site and the complete physician group.

### Analysis

We used Microsoft Excel 2002 (Microsoft Corporation, Seattle, WA) for data entry and simple descriptive statistics after import from the EHR data report. Descriptive statistics for continuous variables were presented using means, quartiles and standard deviations (SD). We presented categorical variables as the percentage of the occurrence frequency with p values generated using chi-square tests and t-tests, as appropriate.

A repeated measures analysis of variance model was used to test for differences in mean rates across three stages. The mixed model included a fixed effect of stage (baseline, stage 2, and stage 3 modeled categorically). We used a general unstructured variance-covariance matrix to account for the correlation of repeated assessments on each participating prescribing physician, and to allow for potentially unequal variances across stages. A Tukey-Kramer multiple comparison procedure was used to compare all pairs of means. We also used statistical inference to determine if the variance parameters were different across the three stages.

## RESULTS

We included for analysis all 47 physicians who remained in the group during the measured interval. Twenty percent of the group’s physicians were non-emergency medicine residency trained. Overall, there were 149,884 eligible patient discharges combined for all practice sites (Stage 1: 82,24; Stage 2: 35,525; Stage 3: 32,118).

**Figure 1:**
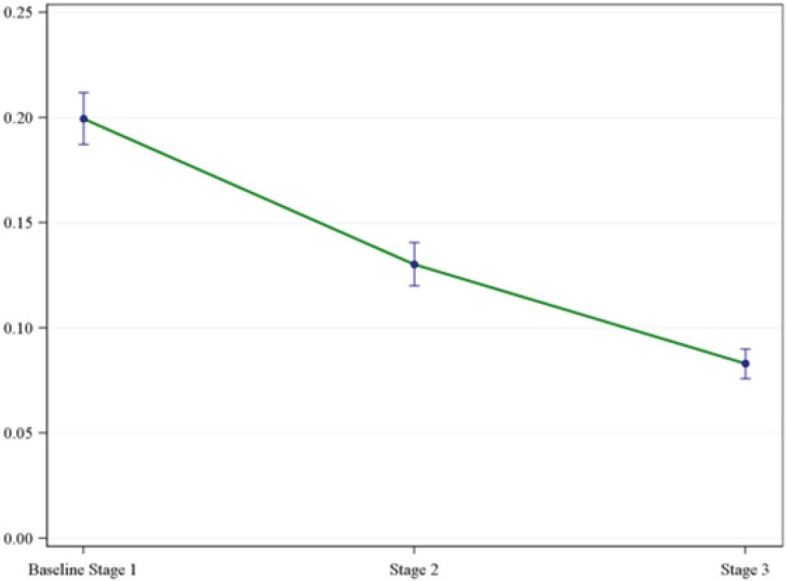
Mean stage physician prescribing rates and corresponding standard errors by stage of intervention.

The variability in physician prescribing decreased through each stage of the initiative, as represented by the distributions of the standard error and standard deviations for each mean rate (Table 1). The overall results of the mixed-effects model for physician opioid prescribing rates suggest there is at least one difference between mean prescribing physician rates among the three separate stages (Figure 1). For a more granular evaluation of the differences in rates means, we applied a Tukey-Kramer multiple comparison procedure to all pairwise comparisons of mean rates. Differences between each pair of mean stage rates were demonstrated (Table 2).

The mean opioid pill count decreased in each stage: 16 pills in Stage 1, 14 pills in Stage 2 (18% reduction, p<0.01), and 13 pills in Stage 3 (18% reduction, p<0.01). The group mean prescribing rate also decreased through each stage. Physician opioid prescribing rate: 20% in Stage 1 (baseline), 13% in Stage 2 (46% reduction, p<0.01), and 8% in Stage 3 (60% reduction, p<0.01) (Table 1).

## LIMITATIONS

External validity for this QI initiative is limited by the use a single physician group in one state. Webster et al. reported that geographic variation in opioid prescribing for acute back pain was associated with state-level contextual factors framed by social conditions such as income, healthcare access and workers compensation.^13^ Our patient population may differ from those at other centers; however, the fundamental concept of reducing prescribing variability in the practice of emergency medicine remains valid.

We chose not to compare practice sites given the overlap of physician practice between each site. In this large emergency medicine practice group, physicians work at different practice locations during the course of each year. Physicians typically have a “base” site with a second practice location occupying up to 50% of their clinical duties. It is uncommon, however, for physicians to practice at more than two sites. Not all physicians in the ED practice group are EM boarded. However, the percentage of emergency medicine board certification is similar to the most recent national emergency physician workforce estimates, and the intervention was directed at all ED physicians.^14^

The before-and-after project design is a limitation in that the validity of the results can be affected by secular trends, i.e. events in addition to the intervention may have caused a change in the outcome being studied. As noted in the methods, there were no changes in the facility or state prescribing guidelines during the data collection period and the PDMP was available and unchanged throughout the QI project. The nature of QI interventions and the necessity of implementing this change systemwide precluded the use of a control group; therefore, we were unable to account for the effects of other variables.

The Hawthorne effect, in which individuals who know they are being observed modify their behavior while such monitoring is in effect, is a recognized limitation of interpreting QI initiatives. We attempted to minimize this during the initial stages by blinding the provider to the objective of the QI (to reduce variability). This phenomenon can actually be harnessed in QI projects to sustain positive changes in practice by committing to continuous and ongoing provider feedback.^15^ The reliability of the reception of individual prescribing information and the individual interpretation of this data was not measured.

In the context of the larger issue of pain management and the role of opioid analgesics, the main limitation of this QI intervention was the chosen outcome of the process (opioid prescribing) rather than patient level outcomes. Measuring patient outcomes (the efficacy of opioid analgesics prescriptions, return visits for pain, patient satisfaction, abuse, misuse, and the long-term impact of this intervention) was outside the scope of this project. We did not link the individual prescriptions to diagnostic information and have no ability to measure the clinical appropriateness of each prescription. Ideally, any assessment of the appropriateness of an opioid analgesic prescription would weigh the risks against the benefit of their intended use. Our QI effort and analysis did not allow for detailed insight into the choices made by specific physicians that changed their prescribing practices during the study intervals. Lastly, we did not address the sustainability of the impact of this intervention beyond the study period.

## DISCUSSION

Emergency physicians have perceptions of “normal” rates of opioid prescribing for ED patients. They also have a perception of how their prescribing practices compare to their peers. It is expected that these estimations influence their clinical decisions to prescribe opioid analgesics. We found that the use of provider-specific feedback to inform these perceptions can influence prescribing decisions and decrease opioid analgesic prescribing variability within an EM peer group.

Social norms are beliefs and perceptions held by individuals regarding their understanding of normal behavior. Normative feedback involves informing individuals of their own behavioral patterns as compared to the behavior of their peers.^16^ This approach is distinct from the use of audit and feedback. Audit and feedback aims to improve practice by prompting providers (feedback) to modify behavior by comparing their past performance (audit) to professional standards or targets.^17^ Professional standards and targets for ED opioid analgesic prescribing are not clearly defined for the treatment of acute pain. Previous studies have shown that the use of normative feedback to correct beliefs regarding social norms can influence an individual’s behavior. For example, Moreira and Latkin, respectively, demonstrated that the frequency of alcohol use in college students and the risk of human immunodeficiency virus transmission in intravenous drug abusers can be reduced through correcting social norms by informing study participants of how their behavior compares to peer behavior.^16^,^18^

Social norms may adversely affect opioid analgesic prescribing decisions if physician estimates regarding their own prescribing behaviors and the prescribing practices of their peers are not accurate. If, for example, a prescriber believes that the “normal” behavior of his or her ED peer group is to prescribe opioids to all patients with low acuity back pain, this will affect the provider’s decision to prescribe. If this assumption is inaccurate, then the decision is inappropriately influenced and the result is an action not consistent with the group. In this investigation, we used a QI initiative to collect prescribing data with normative feedback to accurately inform emergency physicians about their own prescribing rates compared to those of their ED peer group, thus correcting social norm misperceptions.

This QI project is novel in its approach to informing the decision-making process. This intervention starts to address one source of poor quality regarding opioid analgesics: variability in physician opioid prescribing. A previous ED performance improvement program involving a departmental approach to the treatment of dental pain was associated with a reduction in the rate of opioid prescriptions and ED visits for dental pain through the use of a site-specific practice guideline.^18^ In that project, the decreased use of opioid analgesics was accomplished by specifically restricting the use of opioid analgesics for dental diagnoses. Our project did not restrict opioid use or dictate prescribing practices.

Opioid analgesic prescribing to ED patients deemed appropriate for discharge has been relatively understudied. Most efforts to date have been directed at the efficacy, or complications, of specific opioid analgesics or the demographics of patients that may be either over- or under-prescribed analgesics for pain-related ED visits. Deciding whether or not opioids are the safe and appropriate choice for a given patient is wrought with physician preferences and perceptions. We have attempted to address physician perceptions and variability by providing objective prescribing rates.

We observed that sharing of prescribing data among physicians was associated with a decrease in the overall opioid prescribing rate for the physician group. It is possible that these changes in prescribing habits may be due to other factors beyond this initiative or from the fact that they were being tracked (Hawthorne effect). This observation should not necessarily be interpreted as a call or attempt to decrease the number of opioid prescriptions provided to discharged ED patients, as it is not clear that decreased prescribing is the correct target. Indeed, efforts were made from the start of the feedback stages to assure physicians that these data were intended to inform their decision-making and to serve as an opportunity to benchmark their individual practice by site and among their ED peers. It is possible that a reduction in prescribing of opioid analgesics may have been in conflict with the appropriate management of pain in some cases and may not be associated with better long-term outcomes.

Our ultimate goal was to decrease prescribing variability via informed clinician decision-making through the use of open sharing of objective opioid prescribing data that address the possible influence of social norms. Future investigations should evaluate the effect of awareness, and open reporting among peers of physician prescribing rates and opioid pill counts on patient outcomes. This information may serve as valuable internal and external benchmark assessments for emergency medicine prescribing quality.

## CONCLUSION

This QI initiative resulted in reduced variability among ED physicians with respect to frequency of opioid analgesic prescriptions. We also found a decrease in the frequency of opioid prescribing and number of pills per prescription. This low cost intervention that informs opioid prescribing decisions has the potential for wide-reaching impact. Emergency physician opioid prescribing practices and social norm-related prescribing behavior can be influences through the systematic application of prescriber-specific normative feedback and shared peer-prescribing rates.

## Figures and Tables

**Table 1:** Individual stage physician opioid prescribing rate mean with corresponding standard error and standard deviation.

Project stage	Opioid prescribing rate (mean)	Standard error	Standard deviation
1	0.200	0.012	0.084
2	0.130	0.010	0.071
3	0.083	0.007	0.048

**Table 2:** Tukey-Kramer pairwise comparison of physician opioid prescribing rate means between each stage to evaluate for differences between stages.

Stage comparison	Estimate	Adjusted p-value
Baseline to 2	0.069	<0.0001
Baseline to 3	0.117	<0.0001
2 to 3	0.047	<0.0001
